# Effects of Environmental Factors on Concrete Carbonation Depth and Compressive Strength

**DOI:** 10.3390/ma11112167

**Published:** 2018-11-02

**Authors:** Ying Chen, Peng Liu, Zhiwu Yu

**Affiliations:** 1 School of Civil Engineering, Central South University, 22 Shaoshan Road, Changsha 410075, China; cheny83@csu.edu.cn (Y.C.); zhwyu0512@163.com (Z.Y.); 2 National Engineering Laboratory for High Speed Railway Construction, 22 Shaoshan Road, Changsha 410075, China

**Keywords:** concrete, carbonation depth, temperature, relative humidity, CO_2_ concentration, compressive strength

## Abstract

The influence of temperature, CO_2_ concentration and relative humidity on the carbonation depth and compressive strength of concrete was investigated. Meanwhile, phase composition, types of hydration products and microstructure characteristics of samples before and after the carbonation were analyzed by XRD and ESEM. Research results demonstrate that temperature, CO_2_ concentration and relative humidity influence the carbonation depth and compressive strength of concrete significantly. There is a linear relationship between temperature and carbonation depth, as well as the compressive strength of concrete. CO_2_ concentration and relative humidity present a power function and a polynomial function with carbonation depth of concrete, respectively. The concrete carbonation depth increases with the increase of relative humidity and reaches the maximum value when the relative humidity is 70%. Significant differences of phase composition, hydration products and microstructure are observed before and after the carbonation. Carbonization products of samples are different with changes of temperatures (10 °C, 20 °C and 30 °C). The result of crystal structure analysis indicates that the carbonation products are mainly polyhedral spherical vaterite and aragonite.

## 1. Introduction

Factors like environmental or exposure conditions are found to be governing the service life of reinforced concrete structures significantly. One of the major processes influencing the condition of steel bars is carbonation of concrete surrounding steel bars in reinforced concrete structures. Hence, considerable research efforts on the carbonation of concrete in the laboratory and field are carried out around the world [1]. Concrete carbonation is a complex physical and chemical reaction between hydration products of cements and CO_2_. Concrete carbonation easily induces a decrease of the system pH, decomposition of hydration products, depassivation of deactivated film of reinforcing bar and cracking of the concrete cover. Therefore, concrete carbonation is a serious problem for the durability of concrete structures [2,3].

Many achievements on concrete carbonation have been reported in the world [4,5,6]. Lee et al. [7] explored the influence of carbonation on chloride penetration in concrete structures, and the test results indicated that the chloride penetration was more pronounced when the carbonation process was combined with the chloride ingress. Castel et al. [8] investigated the effect of loading on carbonation penetration in reinforced concrete elements, and an accuracy model was proposed to predict the increase of the carbonation depth of the concrete in relation to the tensile stress in rebar. Concrete carbonation is mainly influenced by concrete properties and external environmental factors. All factors interact and restrict mutually and are highly uncertain [9,10]. Existing studies mainly focused on material composition, mixing proportion and carbonation depth of concrete [11,12,13,14]. For example, Zhao et al. [15] studied the effect of the material factors on the carbonation properties of the concrete and proposed the carbonation coefficient values of the concrete mixtures with various material factors. Atis [16] discussed the influence of fly ash, superplasticizer and water to cement ratio on the carbonation of concrete. Lye et al. [17] investigated the influences of crack, freeze-thaw cycling and carbonation on the mechanical properties and durability of reinforced concrete.

Although the analysis of the influences of environmental factors on concrete carbonation has been conducted [18,19,20], existing research conclusions mainly focused on the influences of temperature and CO_2_ concentration on the products, microstructure and depth of concrete carbonation. Ronaldo et al. [11] proposed that microclimatic factors such as temperature and local humidity, sunshine, wind, wetting and drying cycles might have been responsible for the behavior of carbonation in concrete. Cui et al. [21] investigated the relationship between the depth of concrete carbonation and CO_2_ concentration. Andreas et al. [22] discussed the effect of CO_2_ concentration and ambient relative humidity on accelerated and natural carbonation of 18 concrete mixtures produced with nine different cement types, and the results indicated that the water-to-cement ratio and cement-specific effect affected the relative carbonation resistance between the concrete mixtures.

Those above research shortages determine the poor accuracy of the theoretical model of concrete carbonation and the great gap between prediction results and measured results. This makes prediction of the endurance lifetime of actual engineering more apt for the concrete carbonation empirical model of concrete. It is urgent to study the influences of environmental factors on concrete carbonation performances for the sake of better evaluation on concrete endurance, disclosure of the carbonation mechanism and prediction of the service life of the concrete engineering structure.

In this study, the influence of temperature and CO_2_ concentration and relative humidity on concrete carbonation was investigated. Carbonation depth and compressive strength of concrete with different strength grades under different environmental conditions were tested. Moreover, phase composition, as well as types and microstructure of hydration products before and after carbonation were analyzed by X-ray diffraction (XRD) and the environment scanning electron microscope (ESEM).

## 2. Experimental Procedure

### 2.1. Raw Materials

The P_+_O 42.5 Portland cement was provided by China Building Materials Academy. The fly ash produced by Hunan Xiangtan Power Plant was low calcium fly ash (i.e., F class). The physical properties of cement and fly ash are listed in Table 1, and the chemical compositions of fly ash are listed in Table 2. River sands and continuous grading limestone gravels with a grain size of 5–20 mm were collected. Tap water, which met the JGJ63-2006 Water Standards for Concrete, was applied in the stirring process. The polycarboxylic acid series of a high-efficiency superplasticizer, which contained 30% of solid, was from Huangteng Co. Ltd. (Changsha, Hunan, China). Three strength grades of concrete mixing proportions were prepared (Table 3), which was the concrete mix for a lab building’s floor, wall and column.

### 2.2. Experimental Process

Concrete specimens (100 mm × 100 mm × 300 mm and 100 mm × 100 mm × 100 mm) were cast according to GB/T 50082-2009 Standards on the Test Method of Ordinary Concrete Long-term Performance and Endurance. Specimens were demolded after 24 h of casting, and 120 specimens were cast for each concrete grade. Then, they were cured at a temperature of (20 ± 1) °C and a relative humidity of (95 ± 5)% for 28 d. Subsequently, the specimens were dried for 48 h at 60 °C. After drying, the specimens were placed in the environmental simulation test chamber for a week to ensure the same temperature and humidity between the concrete interior and external environment. In order to obtain the carbonation result of concrete in a short time, the high CO_2_ concentration was adopted. Therefore, the corresponding concrete carbonation of this study belonged to the accelerated carbonation test method, and the results were applicable to characterize the change of accelerated carbonation test. Carbonation testing conditions of concrete were acquired by the environmental simulation test system. After the concrete carbonation test reached the preset carbonation age (28 d), the compressive strength and carbonation depth of specimens were tested subsequently. Carbonation depth of concrete was tested by 1% phenolphthalein reagent, which was prepared by 95% ethyl alcohol. Ten points on the cross-section of specimen were measured, and the corresponding average value was set as the carbonation depth with a precision of 0.1 mm. The cement paste with a water to cement ratio of 0.258 was prepared to investigate the hydrations before and after carbonation by XRD analysis. The carbonation conditions of cement paste were the same as the corresponding concrete specimens. Meanwhile, the concrete sample was prepared to carry out ESEM analysis. The mechanical properties of concrete were tested according to the GB/T50081-2002 Standards on the Test Method of Ordinary Concrete Mechanical Properties under the loading rate of 5 kN/s. The average value of three samples was set as the compressive strength of each group.

### 2.3. Test Instruments and Devices

Test instruments mainly included a Quanta-200 environment scanning electron microscope (ESEM) made by FEI Company (Hillsboro, OR, USA), a WAW-DP Universal tester made by Shanghai Sansi Co. Ltd. (Shanghai, China) and the environmental simulation test system made by Wuhan Jinyatai Instrument Co. Ltd. (Wuhan, China). Moreover, BD-86 X-ray diffraction (XRD) produced by Rigaku Company (Akishima, Japan) was used to investigate the hydration products. The corresponding scanning step was 0.02°, and the scanning range was from 5°–60° with a tube voltage and rated power of 60 kV and 4 kW, respectively.

## 3. Results and Discussions

### 3.1. Effects of Temperature on Carbonation Depth and Compressive Strength of Concrete

**Figure 1:**
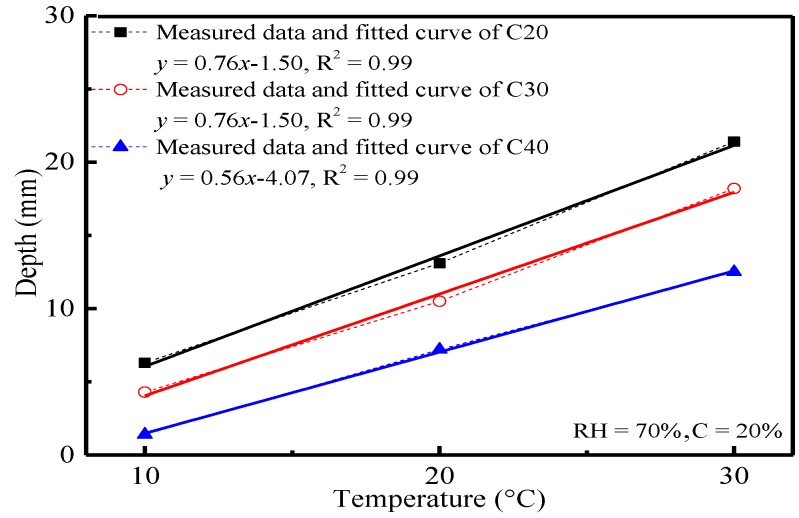
Relation curve between the carbonation depth of concrete and temperature.

**Figure 2:**
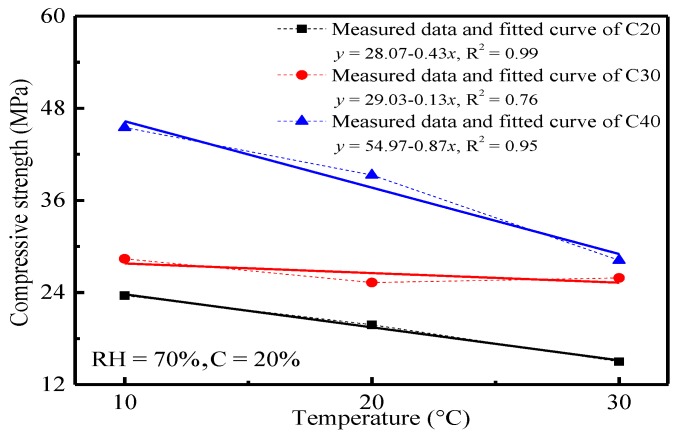
Relation curve between the compressive strength of concrete and temperature.

Generally, concrete carbonation is a complicated physical and chemical reaction. Temperature can influence the chemical reaction significantly. In this paper, the influences of temperature (10 °C, 20 °C and 30 °C) on concrete carbonation were analyzed by the changes of carbonation depth and compressive strength of concrete, which had been carbonized for 28 d under 70% relative humidity and a 20% CO_2_ concentration. The variation curves of carbonation depth and compressive strength of concrete with temperature are shown in Figure 1 and Figure 2, respectively.

It can be seen from Figure 1 that there is a good linear relationship between the carbonation depth of concrete and temperature. Given the same environmental conditions, the carbonation depth of concrete decreased with the increase of the strength grade of concrete. This was because the CO_2_ concentration and carbonation reaction rate in concrete were higher as a response to the high transmission coefficient of CO_2_ and the chemical reaction coefficient under high temperature. Although the carbonation reaction rate of concrete presented a nonlinear relationship with temperature, CO_2_ concentration in concrete took the dominant role, and the whole concrete carbonation was controlled by the transmission of CO_2_. Generally speaking, the transmission coefficient of CO_2_ was closely related to the temperature. Hence, there was a basically linear relationship between the carbonation depth of concrete and temperature. With the increase of concrete strength grade, the microstructure of concrete was manifested by higher density, lower porosity, higher complexity of pore structural characteristics and sinuosity, as well as smaller openness of pores. Combined with Table 2, it can also be seen that the carbonation depth decreased with the decrease of fly ash content. This may be due to more hydration products generated from cement [23,24], so the carbonation resistance was good. As a result, the transmission coefficient of CO_2_ in concrete decreased. If the concrete carbonation process was controlled by CO_2_ transmission in concrete, the carbonation depth of concrete was negatively correlated with concrete strength grade. At the same time, products from the reaction between hydration products and CO_2_ in the concrete surface filled in some pores, which further increased the surface density of concrete and decreased the CO_2_ transmission coefficient in concrete. Hence, the carbonation reaction of concrete declined.

In Figure 2, the compressive strength of concrete decreased with the increase of temperature. A strong linear relationship between them was observed. This could be explained as follows. Concrete carbonation may induce the reaction of CO_2_ with hydration products, such as calcium hydroxide (i.e., CH), calcium silicate hydrate (i.e., CSH), ettringite (i.e., AFt) and calcium aluminate hydrate (i.e., CAH) in concretes, through which gelling hydration products were decomposed and carbonates and calcium silicates without gelling properties were produced. This induced deterioration of the concrete microstructure changes of the hydration products and destroys the chemical balance in the system. Hence, the compressive strength of concrete declined as the carbonation continued. The CO_2_ transmission coefficient and chemical reaction coefficient in concrete increased with the increase of carbonation temperature, which resulted in a higher concrete carbonation degree. Comparing with Figure 1 and Figure 2, the compressive strength of concrete was closely related to the carbonation depth. The higher the carbonation depth is, the sharper the reduction of the compressive strength of concrete will be. This proved indirectly that concrete carbonation may cause changes of the internal microstructure, the decomposition of hydration products and the deterioration of the macroscopic performances of concrete.

### 3.2. Effects of Relative Humidity on the Carbonation Depth and Compressive Strength of Concrete

**Figure 3:**
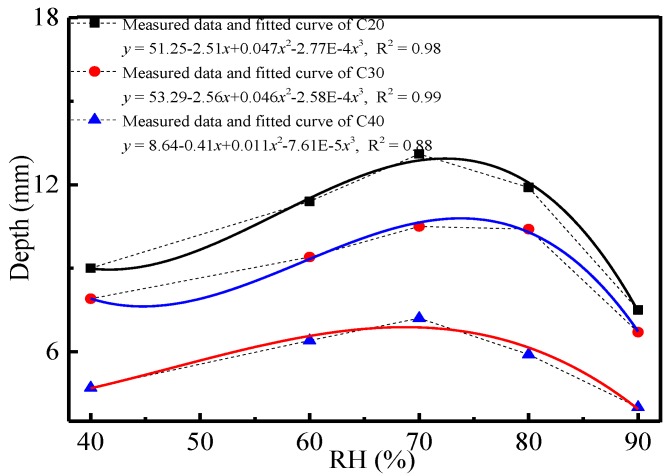
Relationship curve between relative humidity and the carbonation depth of concrete.

**Figure 4:**
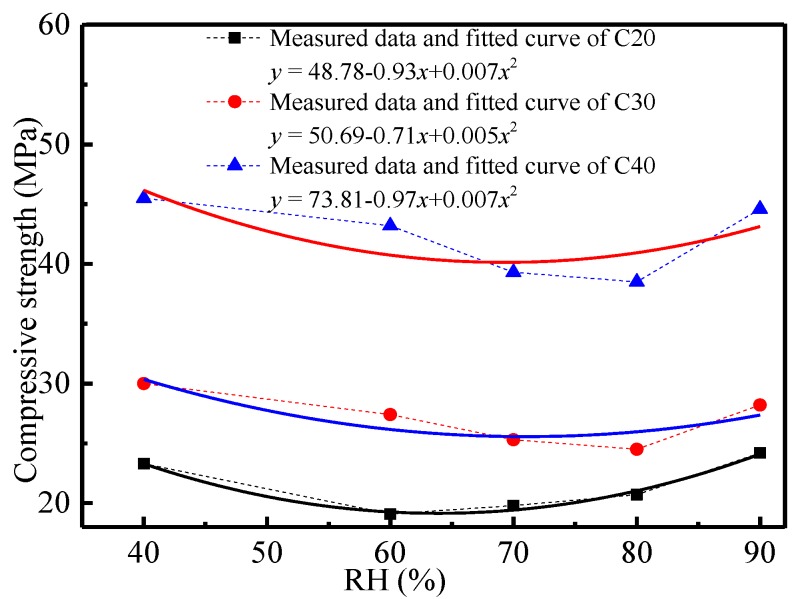
Relation curve between the compressive strength of concrete and relative humidity.

The influencing law of relative humidity on carbonation depth and compressive strength of concrete was analyzed, as shown in Figure 3 and Figure 4.

In Figure 3, relative humidity influenced the carbonation depth of concrete significantly. Specifically, the carbonation depth of concrete increased with the increase of relative humidity and reached the peak when the relative humidity was 70%. The carbonation depth of concrete decreased gradually with the continuous growth of relative humidity. It showed a polynomial function between relative humidity and carbonation depth. This reflected that the concrete carbonation rate and degree were the highest when the relative humidity was 70%. Abundant pores in concrete can be filled in by water vapor and generate liquid water under a certain relative humidity. The water vapor can decrease pore connection in a certain radius of pores. The CO_2_ transmission coefficient in liquid water in concrete pores was very small, which decreased the CO_2_ transmission quantity and rate in concrete accordingly. Therefore, the concrete carbonation depth decreased under relatively high relative humidity (>70%). When the relative humidity was low, water vapor in concrete pores may cover up pore walls and form a liquid film. Different hydration products of cement (mainly CH) were dissolved in the film, and the corresponding oversaturated solution was formed. CO_2_ was transmitted into concrete pores and dissolved in the film to react with CH to generate calcium carbonate. The continuous carbonation consumed abundant ions. This induced decomposition of hydration products in the system and caused further changes of the chemical equilibrium state in the system. If the relative humidity was low, the film area and thickness on the pore wall in concrete were too small to support the carbonation of the system. Therefore, the corresponding concrete carbonation was slow. With the increase of relative humidity, the water content in concrete pores increased, which was conducive to concrete carbonation. Macroscopically, this was manifested by the increase of the carbonation depth of concrete with the increase of relative humidity. The carbonation depth of concrete began to decrease after the relative humidity exceeded a fixed value (about 70%). This deduction could be verified by the changes of the compressive strength of concrete with relative humidity (Figure 4).

Figure 4 shows that the compressive strength of concrete changed with the increase of relative humidity. In a certain range of relative humidity (60–80%), a reduction of compressive strength of concrete reached the extreme value. With the increase of relative humidity, the reduction amplitude of the compressive strength of concrete decreased to some extent. This was because under a certain relative humidity, pores within a certain diameter range in concrete can be saturated by water vapor, which may be transformed into liquid water in pores, which filled some pores in concrete and thereby decreased CO_2_ transmission in concrete. Therefore, carbonation in concrete was dominated by CO_2_ transmission under excessive relative humidity. The corresponding carbonation depth of concrete was relatively low, and the reduction of compressive strength under high humidity was relatively small. Nevertheless, the liquid water content in concrete pores was positively related to relative humidity in a certain range (<70%). It could offer places for dissolution and reaction of hydration products (e.g., CH), which increased carbonation depth and rate accordingly. As the carbonation continued, microstructure and hydration products of the system changed, which were manifested by a reduction of the compressive strength of concrete macroscopically.

### 3.3. Effects of CO_2_ on the Carbonation Depth and Compressive Strength of Concrete

**Figure 5:**
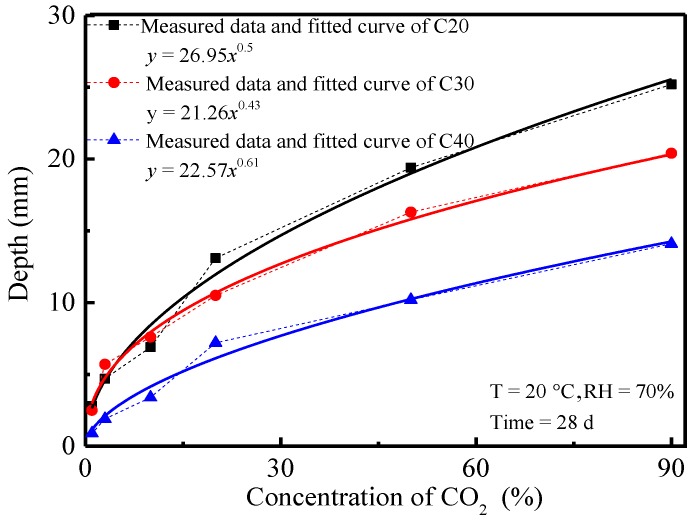
Relation curves between the carbonation depth of concrete and CO_2_ concentration.

**Figure 6:**
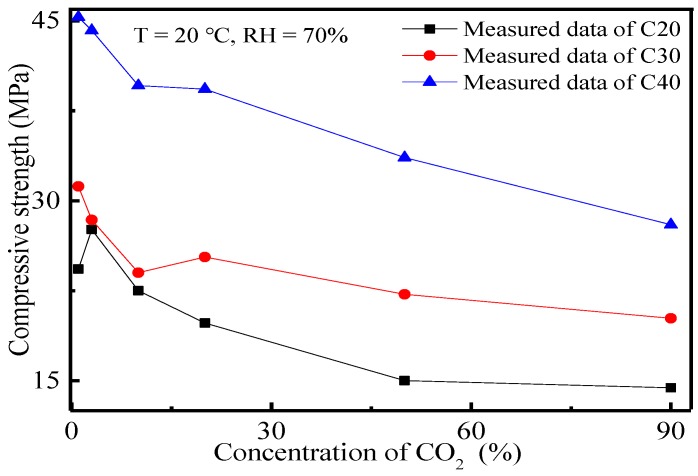
Relation curves between the compressive strength of concrete and CO_2_ concentration.

The effects of CO_2_ concentration on the carbonation depth and compressive strength of concrete were also investigated. The variation curves of carbonation depth and compressive strength of concrete at 28 d and the CO_2_ concentration are shown in Figure 5 and Figure 6.

In Figure 5, carbonation depth of concrete under the same carbonation conditions increased with the increase of CO_2_ concentration. A power function between them was observed. Under the same CO_2_ concentration, carbonation depth of concrete was negatively correlated with the strength grade of concrete. Given the higher CO_2_ concentration, the inner-outside concentration gradient was larger, and more CO_2_ could diffuse into the concrete and react with the hydration products in concrete. The growth rate of carbonation depth decreased as CO_2_ concentration increased. This was because there was a low CO_2_ concentration in the early carbonation of concrete and the CO_2_ quantity transmitted into concrete was consumed by a fast reaction. As the CO_2_ concentration increased, the CO_2_ quantity transmitted into the concrete was higher than the CO_2_ consumption by concrete carbonation. Concrete carbonation was controlled by chemical reaction rather than the CO_2_ transmission. As a result, the change rate of the concrete carbonation depth curve decreased gradually with the increase of CO_2_ concentration. A high strength grade of concrete implied a higher density, lower porosity and more closed pores in concrete, as well as the lower CO_2_ transmission coefficient in concrete. Hence, CO_2_ quantity transmitted into concrete decreased accordingly. Moreover, a higher strength grade of concrete demanded greater cement consumption and more hydration products in the hydration reactions, which helped the system to absorb more CO_2_. Therefore, the carbonation depth of concrete was negatively related to the strength grade of concrete. This could be verified by the relation curves between the compressive strength of concrete and CO_2_ concentration. Changes of the compressive strength of concrete after 28 d of carbonation under different CO_2_ concentrations are shown in Figure 6. Temperature and relative humidity were set to 20 °C and 70%, respectively.

It can be seen from Figure 6 that the compressive strength of concrete decreased with the increase of CO_2_ concentration. Under the same test conditions, the influences of the carbonation effect on concrete strength intensified in response to the decreasing strength grade of concrete. Since concrete carbonation was the reaction between gelling hydration products (e.g., CH, CSH, CAH and AFt) and CO_2_, hydration products were decomposed and decreased. The system microstructure was loosened and deteriorated accordingly, thus decreasing the compressive strength of concrete. With the increase of CO_2_ concentration, the CO_2_ concentration gradient inside and outside of the concrete surface, as well as the CO_2_ quantity transmitted into the concrete increased. Therefore, there was more CO_2_ participating in the reaction and decomposition of the abundant hydration products that caused the microstructure failure of concrete. Macroscopically, this was manifested by a reduction of the compressive strength of concrete. For C20 concrete, a low CO_2_ concentration may increase the concrete strength. This might be caused by CO_2_ reacting with the hydration products in the concrete surface. The reaction products were generated and filled in some part of the pores, which enhanced the density of the concrete microstructure. The positive effect of carbonation in a short period was caused by the dominant role of the concrete microstructure.

### 3.4. XRD and ESEM Analysis before and after the Concrete Carbonation

**Figure 7:**
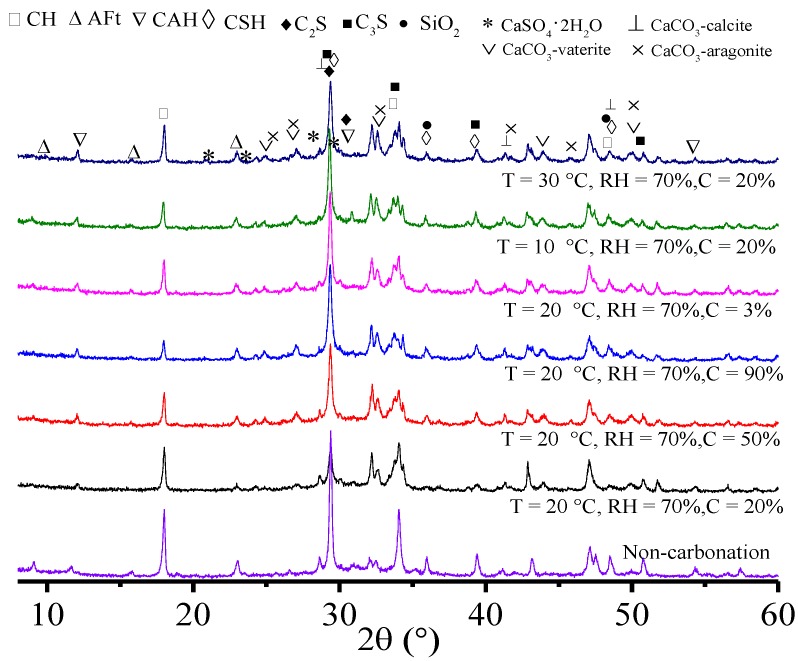
XRD spectra of the phase composition before and after carbonation.

The types of hydration products and the phase composition of specimens before and after the carbonation were analyzed to disclose the carbonation mechanism of concrete and its performance changes. The XRD spectra of the phase composition before and after the carbonation are shown in Figure 7.

It can be seen from Figure 7 that there was a significant difference in the XRD spectra before and after the carbonation, which manifested as the intensity, disappearance and generation of the diffraction peak. The diffraction peak of hydration products of non-carbonation specimens demonstrated that CH, AFt, CSH and CAH were major hydration products. However, the diffraction peaks of different crystal styles of calcium carbonate (i.e., calcite, vaterite and aragonite) were observed when the specimens were carbonized, which implied that the carbonation product was calcium carbonate. There were significant differences in the phase composition and products of specimens when the temperature and CO_2_ concentration were different. These were mainly manifested by the attenuation (e.g., CH, CSH and CAH) and disappearance (e.g., AFt) of the diffraction peaks of some cement gelling hydration products, as well as the appearance and strengthening of the diffraction peaks of carbonation products (e.g., CaCO_3_). When the temperature and relative humidity were fixed, the diffraction peak intensity of CaCO_3_ was positively related to CO_2_ concentration. The CaCO_3_ content in carbonized samples increased. This was because more and more CO_2_ may be transmitted into samples and react with hydration products in the system under the high CO_2_ concentration. Figure 7 also reflected that under the same CO_2_ concentration and relative humidity, the phase composition and diffraction peaks of hydration products changed more significantly with the increase of temperature. This was caused by the faster decomposition of the carbonation products and hydration products of the system under the higher temperature.

**Figure 8:**
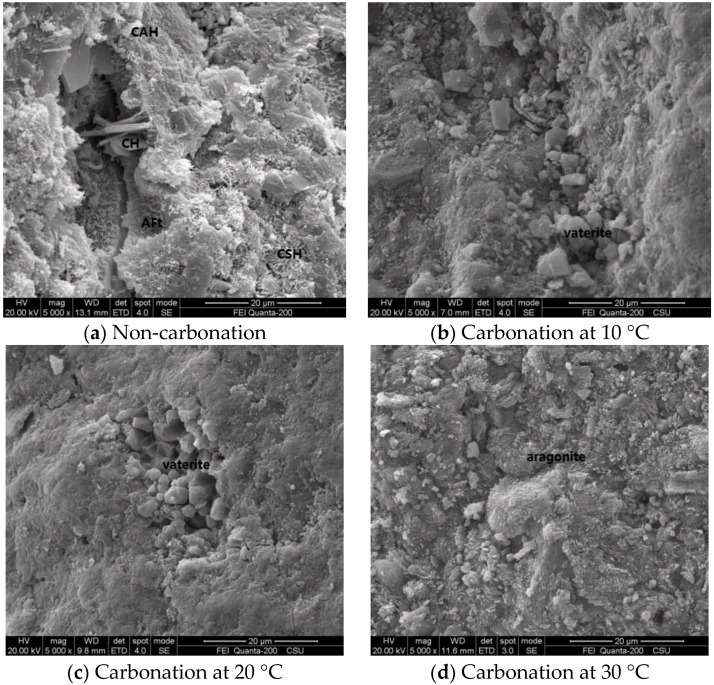
ESEM spectra of carbonation specimens under different temperatures.

The microstructure and product morphology before and after the carbonation were studied to investigate the influences of temperature on concrete carbonation. The ESEM spectra of specimens before and after the carbonation under different temperature are shown in Figure 8.

In the ESEM spectra of non-carbonation samples, the hydration products were mainly hexagonal plate-like CH, rod-like AFt, flocculent CSH and CAH (Figure 8a). Nevertheless, there were few gelling hydration products in the microstructure of carbonized specimens and particulate CaCO_3_ in the pores. The carbonation products of specimens under different temperatures were also different to some extent. Specifically, polyhedral vaterites could be observed in the ESEM spectra of carbonized samples under 10 °C and 20 °C (Figure 8b,c). Under 30 °C, the microstructure and morphology of the hydration products of carbonized samples were significantly different from those under 10 °C and 20 °C (Figure 8d). Hexagonal plate-like CH, arborization CSH, flocculent CAH and rod-like AFt disappeared, and abundant aragonites were generated, which implied the decomposition and destruction of the original cementitious hydration of cement. These phenomena were the consequence of thermodynamic equilibrium states under different temperatures. The chemical reaction between CO_2_ and hydration products in the system took place under different temperatures, which produced CaCO_3_ crystals of different forms.

## 4. Conclusions

1 Temperature, CO_2_ concentration and relative humidity influence the carbonation depth and compressive strength of concrete significantly. Temperature has a linear relationship with the carbonation depth and compressive strength of concrete. CO_2_ concentration and relative humidity present power and polynomial functions with the carbonation depth of concrete, respectively.
2 The carbonation depth of concrete is positively correlated with temperature and CO_2_ concentration, but the compressive strength of concrete is negatively correlated with the strength grade of concrete. The carbonation depth of concrete increases with the increase of the relative humidity and reaches the peak when the relative humidity is 70%. This is because the CO_2_ transmission coefficient and chemical reaction coefficient may increase with temperature. Besides, the increase of the CO_2_ concentration may bring the increase of the concentration gradient and the CO_2_ concentration in concrete, as well as the intensified carbonation. Concrete density is positively related to the strength grade of concrete. The CO_2_ transmission coefficient in concrete is low, which may decrease the carbonation depth of concrete.
3 The phase composition, hydration products and microstructure of concrete change significantly before and after the carbonation. Such changes are mainly manifested by the disappearance and attenuation of the diffraction peak of some hydration products. XRD and ESEM spectral analysis reveal that hexagonal plate-like CH, rod-like AFt, flocculent CSH and CAH are major hydration products before the carbonation, but particulate CaCO_3_ takes the dominant role in hydration product composition after the carbonation. Temperature affects the crystal form of carbonation products. Polyhedral spherical vaterites are major carbonation products under 10 °C and 20 °C, whereas aragonites are the major carbonation products under 30 °C.

## Figures and Tables

**Table 1:** Physical properties of cement and fly ash.

Items	Fineness (74-μm mesh) (%)	Density (g/cm^3^)	Specific Surface Area (m^2^/kg)	Water Requirement of Normal Consistency (%)	Initial Setting Time (min)	Final Setting Time (min)	Compressive Strength (28 d) (MPa)
Cement	0.6	3.15	349	25.8	130	195	45.8
Fly ash	1.5	2.83	322	-	-	-	-

**Table 2:** Chemical compositions of fly ash (%).

CaO	SiO_2_	Al_2_O_3_	Fe_2_O_3_	MgO	Na_2_O	K_2_O	SO_3_	P_2_O_3_	Loss
2.57	54.0	27.7	6.11	1.23	0.37	1.50	0.14	-	2.56

**Table 3:** Mix of concrete (kg/m^3^).

Items	Cement	Fly Ash	Fine Aggregate	Coarse Aggregate	Water	Water Reducer
C20	195	128(39.6%)	785	1045	178	1.8
C30	270	125(31.6%)	780	1050	172	1.9
C40	350	122(25.8%)	710	1052	162	2.25

Note: The values in bracket are the ratio of fly ash to total amount of cementitious materials.
